# Effectiveness of a Ligating Device for Endoscopic Surgery

**DOI:** 10.1155/DTE.2.47

**Published:** 1995

**Authors:** T. Hachisu, H. Yamada, K. Hamaguchi

**Affiliations:** 1 Department of Surgery Sakura National Hospital 2-36-2 Ebaradai Chiba Prefecture Sakura 285 Japan; 2 Department of Pathology Sakura National Hospital Japan

## Abstract

A new detachable snare for hemostasis in the removal of large polyps or other elevated lesion was developed by the author (Olympus Ligating Device). It allows ligation to be performed through the channel of an endoscope using a nonconductive loop that can be detached from the ligator. At Sakura National Hospital, endoscopic ligation with this device was performed in 80 patients from May 1989 to January 1994. The purpose of the procedure was preventive hemostasis prior to the endoscopic resection of large elevated lesions in 71 patients and for control of hemorrhage in 9 patients. The elevated lesions were polyps in 69 patients and submucosal tumor in 2, being pedunculated in 36 and semipedunculated in 35. The maximum diameter of these lesions ranged from 15 to 40 mm (mean: 23 mm), being greater than 20 mm in 57 cases. The 9 patients undergoing endoscopic ligation for hemorrhage had bleeding polypectomy stumps (n = 5), bleeding polyps (n = 3), and a bleeding esophageal varix (n = 1). Endoscopic ligation achieved the complete prevention of hemorrhage following the resection of elevated lesion in 63/71 patients (88.7%) and, in combination with a HX-3L clip, allowed endoscopic resection to be performed in 70/71 patients (98.6%). In the 9 patients with bleeding lesions, complete hemostasis was achieved without complications.


*Diagnostic and Therapeutic Endoscopy*, 1995, Vol. 2, pp. 47–52
Reprints available directly from the publisher
Photocopying permitted by license only

© 1995 Harwood Academic Publishers GmbH
Printed in Singapore

### Effectiveness of a Ligating Device For Endoscopic Surgery

T. HACHISU,[1] H. YAMADA and K. HAMAGUCHI[2]

[1]*Department of Surgery, Sakura National Hospital, 2-36-2 Ebaradai, Sakura, Chiba, 285 Japan;*
[2]*Department of Pathology, Sakura National Hospital*

*(Received August 15, 1994; in final form February 24, 1995)*

A new detachable snare for hemostasis in the removal of large polyps or other elevated lesion was developed by the author (Olympus Ligating Device). It allows ligation to be performed through the channel of an endoscope using a nonconductive loop that can be detached from the ligator. At Sakura National Hospital, endoscopic ligation with this device was performed in 80 patients from May 1989 to January 1994. The purpose of the procedure was preventive hemostasis prior to the endoscopic resection of large elevated lesions in 71 patients and for control of hemorrhage in 9 patients. The elevated lesions were polyps in 69 patients and submucosal tumor in 2, being pedunculated in 36 and semipedunculated in 35. The maximum diameter of these lesions ranged from 15 to 40 mm (mean: 23 mm), being greater than 20 mm in 57 cases. The 9 patients undergoing endoscopic ligation for hemorrhage had bleeding polypectomy stumps (n = 5), bleeding polyps (n = 3), and a bleeding esophageal varix (n = 1). Endoscopic ligation achieved the complete prevention of hemorrhage following the resection of elevated lesion in 63/71 patients (88.7%) and, in combination with a HX-3L clip, allowed endoscopic resection to be performed in 70/71 patients (98.6%). In the 9 patients with bleeding lesions, complete hemostasis was achieved without complications.

**KEY WORDS:** clipping device, detachable snare, endoscopic ligation, HX-3L clip, ligating device

### INTRODUCTION

Conventional surgery and endoscopic surgery have many features in common, and conventional surgical techniques can often be utilized during endoscopy after some modifications. For example, high frequency current, microwave, and laser are commonly used in conventional surgery, and are also effective in endoscopic surgery. Corresponding to the hemo-clip or the surgical suture used in conventional surgery, an endoscopic clipping device was developed by Hayashi (1) and Kuramata (2) in 1975. The current version was developed by the author in 1988 (3). This device is widely used in endoscopic surgery for hemostasis (4), marking of lesions (5), ligation of varices (6), and mucosal suture. If endoscopic ligation with a suture thread were possible, it could be utilized for a wide variety of purposes. Accordingly, the author, in cooperation with Olympus Optical Co. (Tokyo, Japan), developed a detachable snare (ligating device) in 1989 (7). Subsequent modifications led to the development of a more manageable apparatus in 1993. Using this ligating device, gastrointestinal lesions can be surrounded by a nonconductive loop, which is then tightened and detached from the device. With this device, it is possible to prevent hemostasis prior to an endoscopic removal of large elevated lesions. In addition, this technique can control various types of bleeding lesions. In this article, we demonstrate the features and applications of this ligating device and the outcome of endoscopic ligation in 80 patients treated at Sakura National Hospital from May 1989 to January 1994. This paper also discusses the usage of the ligating device alone, and the possibility to use it in combination with the HX-3L clip for control of hemorrhage in endoscopic surgery.

### SUBJECTS AND METHODS

Our original ligating device was designed for the endoscopic ligation of lesions through the working channel of the endoscope. It is composed of a loop that is tightened around the lesion and a ligator (Fig. 1). The ligator consists of a Teflon tube (2.5 mm in diameter), a stainless

Address for correspondence: Tadashi Hachisu, M.D., Department of Surgery, Sakura National Hospital, 2-36-2 Ebaradai, Sakura City, Chiba Prefecture, 285 Japan.

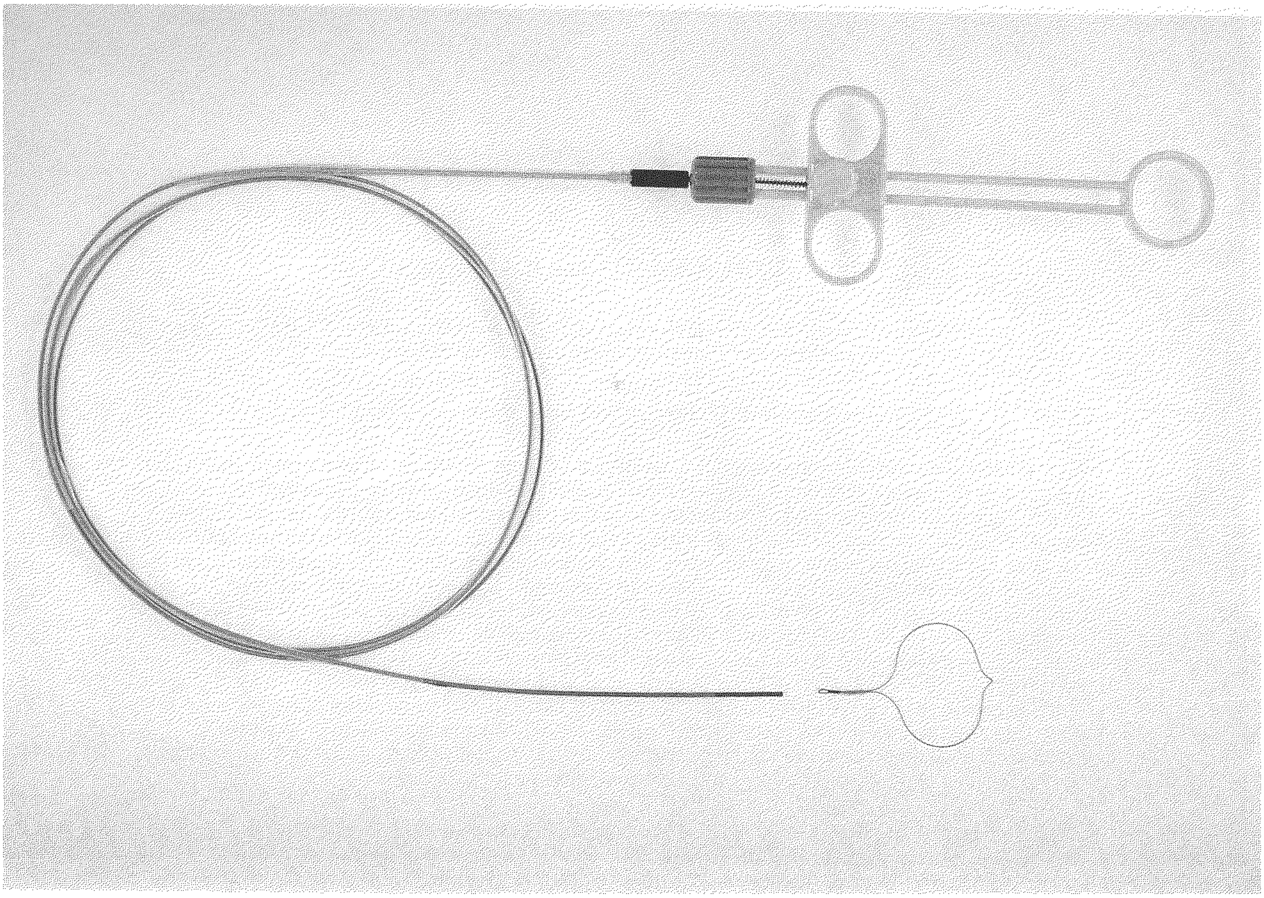

**Figure 1** The ligating device is composed of a ligator and a loop.

steel coil, a hooked wire, and a handle. This apparatus can be used through any endoscope with a 2.8 mm or wider channel. Three models are available with working lengths of 165, 195, and 230 cm, for upper gastrointestinal endoscopes, medium-length colonoscopes, and full-length colonoscopes, respectively. The loop is nonconductive and consists of a heat-treated elliptical nylon thread equipped with a silicone-rubber stopper that maintains the tightness of the loop.

The prototype loop had several problems that have been overcome with the current version (Fig. 2). Thus, the shape and tightness of the loop can now be securely maintained. An experimental study showed that the loop could be used to tightly ligate elevated lesions measuring up to 4 cm in maximum diameter, completely blocking blood flow into the lesion. The loop eventually falls off the bowel wall, with the lesion, into the gut lumen and is spontaneously discharged in the feces. However, if spontaneous discharge is thought to be problematic for any reason, the redundant part of the loop can readily be cut off and removed using an endoscopic suture cutter.

The procedure of endoscopic ligation with this apparatus is illustrated in Figure 3. First, the tube sheath is pulled back to extend the loop. Then the loop is placed around the target lesion and tightened by pulling the slider towards the ring. Finally, the slider is pulled out to the maximum possible extent to detach the loop from the device.

We performed endoscopic ligation with this device in 80 patients from May 1989 to January 1994 for preventive hemostasis prior to the resection of large elevated lesions (n = 71) or to control gastrointestinal bleeding (n = 9). These consisted of 33 females and 47 males aged from 30 to 82 years (mean: 59.3 years). Some patients had serious underlying conditions, including chronic renal failure requiring hemodialysis in 9, liver cirrhosis complicated by severe hepatic dysfunction in 3, and 1 case of Peutz-Jegher's syndrome with a history of four operations for bowel obstruction. The location of the lesions and the reason for using the ligating device in the 80 patients are shown in Table 1.

Of the 71 patients treated by preventive hemostasis, 69 had polyps and 2 had submucosal tumors. Thirty-six of

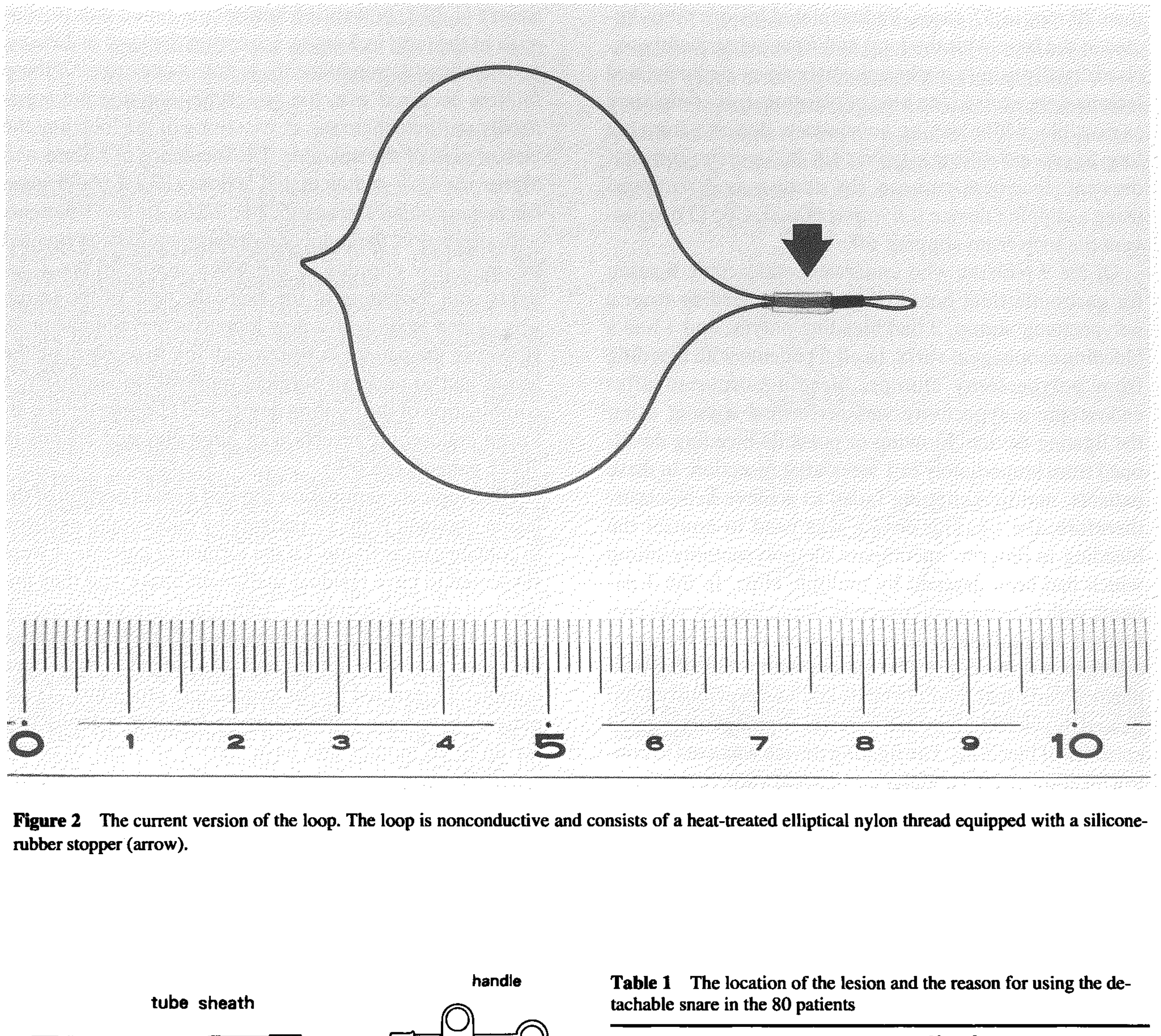

**Figure 2** The current version of the loop. The loop is nonconductive and consists of a heat-treated elliptical nylon thread equipped with a silicone-rubber stopper (arrow).

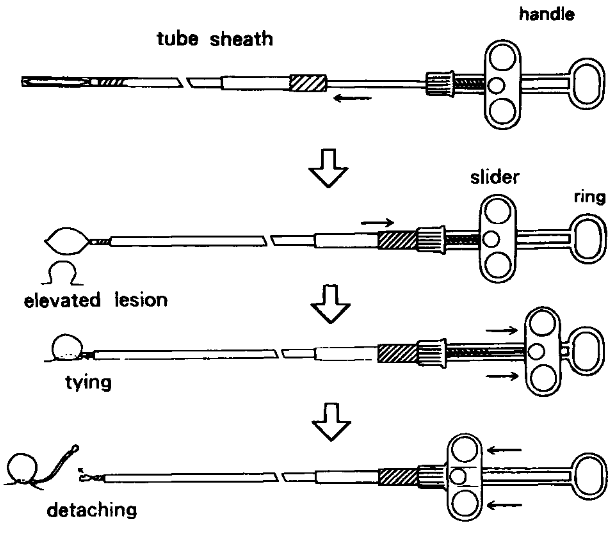

**Figure 3** Endoscopic ligation with the ligating device.

**Table 1** The location of the lesion and the reason for using the detachable snare in the 80 patients

| | *No. of cases* | |
|---|---|---|
| *Location* | *Preventive ligation* | *Hemostatic ligation* |
| Esophagus | 0 | 1 |
| Stomach | 16 | 1 |
| Duodenum | 3 | 1 |
| Ascending Colon | 8 | 0 |
| Transverse Colon | 2 | 1 |
| Descending Colon | 2 | 0 |
| Sigmoid Colon | 37 | 4 |
| Rectum | 3 | 1 |
| Total | 71 | 9 |

the elevated lesions were pedunculated, while 35 (including the 2 submucosal tumors) were semi-pedunculated. The maximum diameter of the elevated lesions ranged from 15 to 40 mm (mean: 23 mm), and was greater than 20 mm in 57 cases. Pedunculated lesions were ligated at the base with the loop and were subsequently excised distally using a diathermic snare. Semi-pedunculated lesions were excised (en bloc or piecemeal) as completely as possible, while leaving an adequate distance from the loop to prevent it from slipping off. In 5 patients who underwent piecemeal excision, the stump was also clipped using a clipping device (Olympus HX-3L Clip (5)) to prevent the loop from slipping off.

Of the 9 patients who underwent endoscopic ligation for gastrointestinal hemorrhage, 5 had bleeding from a polypectomy stump, 3 had bleeding polyps, and 1 had a bleeding esophageal varix. In all 5 patients with bleeding from polypectomy stumps, bleeding occurred after endoscopic polypectomy was performed without using the ligating device. Flowing or pulsatile bleeding developed from immediately to 1 week after resection. In these patients, multiple clipping failed to achieve hemostasis, therefore, the ligating device was used to control the bleeding in order to encompass the polypectomy stump which had been grasped by multiple clips. In the 3 patients with bleeding polyps, endoscopic ligation was performed immediately after detection of the lesion at emergency endoscopy. The patient with a bleeding esophageal varix had liver cirrhosis and had previously been treated by sclerotherapy and variceal clipping for repeated episodes of bleeding. The ligating device was used to control bleeding from a nodular esophageal varix in the lower esophagus.

The redundant part of the loop was cut and removed using an endoscopic suture cutter in the patient with Peutz-Jegher's syndrome who was treated for a large gastric polyp and had a history of four operations for bowel obstruction. This was also done in 3 other patients treated for gastric polyps who had chronic renal failure and severe constipation. However, this additional procedure was not performed in the remaining 76 patients without gastrointestinal complications. The endoscopes used included front-viewing panendoscopes (GIF-Q20, GIF-XQ200, GIF-Q200, Olympus Optical Co.) and colonoscope (CF-20HI and CF-IT200I, Olympus Optical Co.). All patients gave informed consent to treatment with the ligating device after receiving information on its design, therapeutic necessity, previous clinical experience, and the potential risks involved.

### RESULTS

Postoperative hemorrhage was completely prevented in 63 of the 71 patients (88.7%) treated for preventive hemostasis using the ligating device. Among the other 8 patients (11.3%), preventive hemostasis failed due to slippage of the loop in 5 cases, loosening the loop in 2 cases, and inability to strangulate the lesion in one case. All these failures occurred with the prototype loop and were primarily attributable to the shortcoming of the loop and the lack of skill of the operator. The incidence of failure was higher for semi-pedunculated lesions (17.1%; 6/35) than for pedunculated lesions (5.7%; 2/36). In the 5 patients with slippage of the loop, hemorrhage could be prevented by immediate clipping of the polyp stump. In the 2 patients with loosening of the loop, the base of the lesion was ligated again with a new loop to achieve hemostasis. However, the patient in whom inability to strangulate the lesion had to undergo surgical removal. In summary, a combination of the ligating device and clipping device allowed endoscopic resection of large elevated lesions in 70/71 patients (98.6%).

Histological examination showed a small portion of the lesion obtained from 17 patients by endoscopic excision to contain cancer tissue. Of the former 17 patients, 4 were suspected to have residual malignant tumor and thus underwent surgical operation. Additional surgical operation was not performed in the remaining 13 patients because the tumor was very limited and was judged to have been completely resected by the endoscopic procedure. These patients have been followed up by frequent colonoscopy for up to 26 months and have shown no signs of recurrence. Of the submucosal tumors, the histological diagnosis was gastric leiomyoma in one case and lipoma of the sigmoid colon in the other case. In the patient with leiomyoma, endoscopic resection was followed by surgical removal because the possibility of leiomyosarcoma could not be ruled out.

Complete hemostasis was achieved in all 9 patients treated for gastrointestinal hemorrhage using the ligating device (Table 2).

In all 79 patients undergoing endoscopic ligation using the ligating device, the loop detached spontaneously and was discharged in feces without causing any complications, generally within 4–7 days (5.3 days on average) after the ligation procedure. Endoscopy performed after detachment of the loops showed a residual shallow ulcer

**Table 2** Results in 80 cases treated by the ligating device between May 1989 and January 1994

| *Purpose* | *No. of cases* | *No. of effective cases (Effective percentage)* |
|---|---|---|
| Preventive ligation | 71 | 63 (88.7%) |
| Hemostatic ligation | 9 | 9 (100.0%) |
| Total | 80 | 72 (90.0%) |

with complete elimination of the elevated lesion. The ulcers were much smaller and shallower and healed more rapidly than those occurring after endoscopic excision with a diathermy snare alone.

### DISCUSSION

Ligation with a suture thread is one of the most basic surgical techniques. It is frequently used for preventive or therapeutic hemostasis prior to or after the resection of lesions. Ligation is also extremely effective in endoscopic surgery. The ligating device used in this study allows endoscopic ligation through the working channel of the endoscope, and has similar applications to those of conventional surgical ligation. Prior to surgical resection of a large elevated lesion, the base of the lesion is ligated to obstruct the blood supply. Subsequently, the lesion is excised distal to the ligature to prevent hemorrhage (Fig. 4). In a similar way, during endoscopic resection of a large gastrointestinal polyp, ligation of the base of the polyp using the ligating device prior to distal excision with a diathermy snare can prevent hemorrhage. It can also reduce the adverse effects of hyperthermic coagulation of high frequency current on the ligated site and thus reduce the risk of postoperative bowel perforation (Fig. 5). In clinical use, it was possible to prevent hemorrhage following endoscopic resection of large elevated lesions by prior ligation in 63 out of 71 patients (88.7%). When combined with clipping, the ligating device allowed successful endoscopic resection of large elevated lesions in 70 out of 71 patients (98.6%). Resection after ligation left a small ulcer following detachment of the loop, much shallower and smaller than the ulcers formed after usual resection without the ligating device, thus reducing the risk of postoperative perforation.

In conventional operations, ligation of a non-elevated lesion is performed using two clamps to grasp the circumference of the lesion (Fig. 6). This technique is similar to that used to obtain hemostasis of the stump following polypectomy in our series, i.e., the ligating device is used to control the bleeding by completely encompassing the polypectomy stump which had been grasped by multiple clips (Figs. 7–8). This endoscopic technique was also very effective in achieving hemostasis.

Recently, this ligating device has been used extensively for prevention of bleeding prior to polypectomy at many Japanese institutions with favorable results. In a new application of the device, Yoshida et al. (8) reported an excellent outcome in the treatment of gastric varices using a modified stainless steel loop in 1994. They demonstrated that complete disappearance of varices was achieved in 10 out of 10 patients (100%) using this device. This was consistent with the successful hemostasis achieved in our patient with a bleeding esophageal varix. Although recent progress in endoscopic surgery has been remarkable, there is still much need for improvement in terms of apparatus. The ligating device used in this study has the potential for various applications in endoscopic surgery.

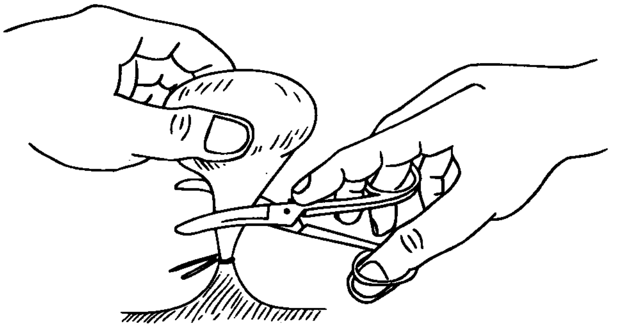

**Figure 4** The conventional surgical resection of a large elevated lesion. The base of the lesion is ligated to obstruct the blood supply. Subsequently, the lesion is excised distal to the ligature to prevent hemorrhage.

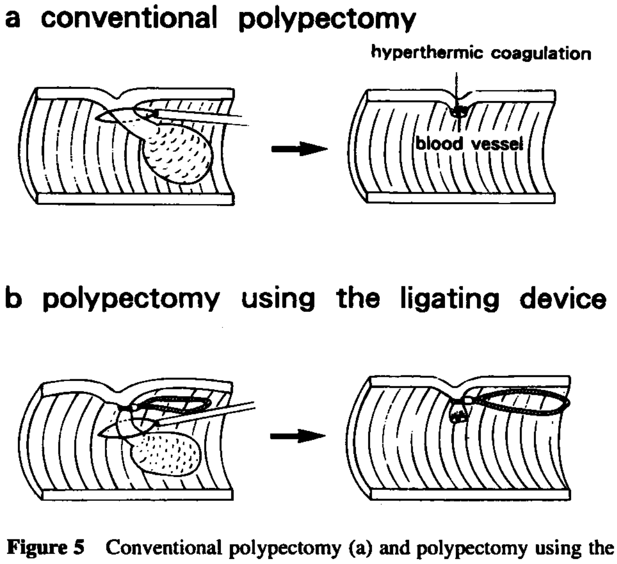

**Figure 5** Conventional polypectomy (a) and polypectomy using the ligating device (b). Endoscopic ligation can prevent hemorrhage and can also reduce the adverse effect of hyperthermic coagulation of high frequency current on the ligating site and thus reduce the risk of postoperative bowel perforation.

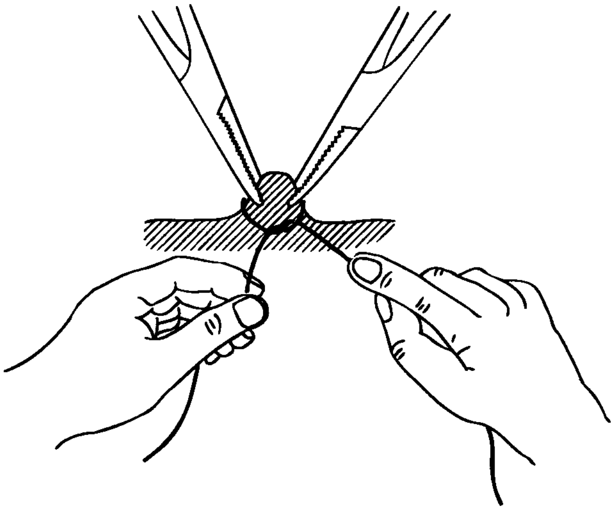

**Figure 6** The conventional surgical ligation of a non-elevated lesion. The ligation is performed using two clamps to grasp the circumference of the lesion.

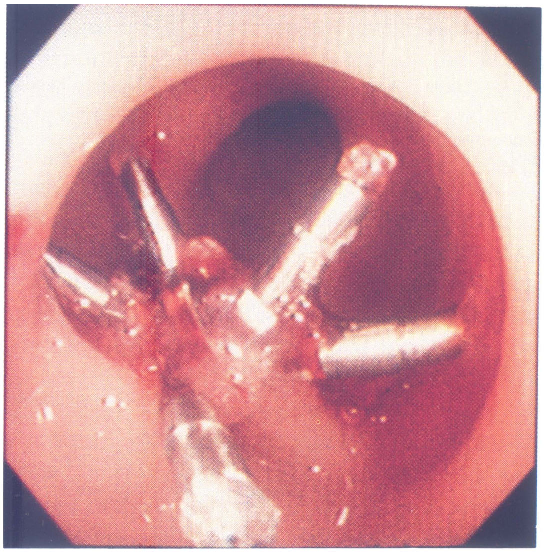

**Figure 7** A case bleeding from a polypectomy stump. In this case, multiple clipping failed to achieve hemostasis.

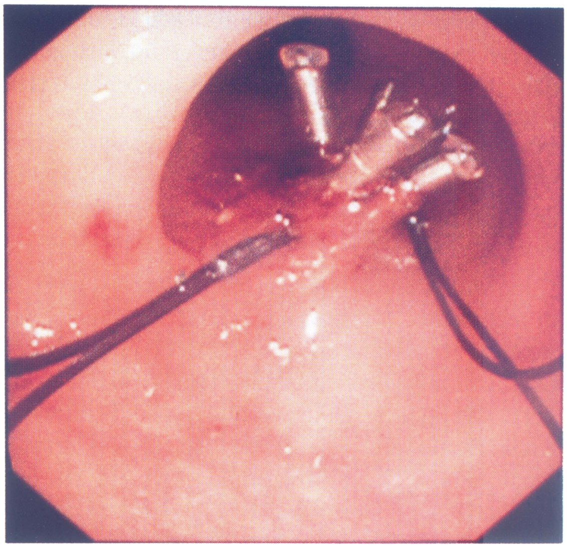

**Figure 8** The ligating device was used to control the bleeding in order to encompass the polypectomy stump which had been grasped by multiple clips.

### REFERENCES

1. Hayashi T, Yonezawa M, Kuwabara T, et al. The study on stanch clip for the treatment by endoscopy. Gastroenterol Endosc 1975;17:92–101.
2. Kuramata H, Etoh S, Horiguchi K, et al. The clinical applications of K-type clip device. Gastroenterol Endosc 1975;17:276 (Japanese abstract).
3. Hachisu T. Evaluation of endoscopic hemostasis using an improved clipping apparatus. Surg Endosc 1988;2:13–17.
4. Binmoeller KF, Thonke F, Soehendra N. Endoscopic hemoclip treatment for gastrointestinal bleeding. Endosc 1993;25:167–170.
5. Hachisu T, Miyazaki S, Hamaguchi K. Endoscopic clip-marking using the newly developed HX-3L, clip. Surg Endosc 1989; 3:142–147.
6. Miyoshi H, Shinkata J, Tokura Y. Endoscopic clipping of esophageal varices. Digestive Endosc 1992;4:147–150.
7. Hachisu T. A new detachable snare for hemostasis in the removal of large polyps or other elevated lesions. Surg Endosc 1991; 5:70–74.
8. Yoshida T, Hayashi N, Suzuki N. et al. Endoscopic ligation of gastric varices using a detachable snare. Endoscopy 1994; 26:502–505.

